# Lay Definitions of Intelligence, Knowledge, and Memory: Inter- and Independence of Constructs

**DOI:** 10.3390/jintelligence11050084

**Published:** 2023-04-28

**Authors:** Jennifer H. Coane, John Cipollini, Talia E. Barrett, Joshua Kavaler, Sharda Umanath

**Affiliations:** 1 Department of Psychology, Colby College, Waterville, ME 04901, USA; 2 Department of Psychological Science, Claremont McKenna College, Claremont, CA 91711, USA

**Keywords:** intelligence, memory, knowledge, metacognition, face validity

## Abstract

The present study examined how lay participants define the following concepts used widely in psychology: being intelligent, knowing, and remembering. In the scientific community, knowledge overlaps with the contents of semantic memory, crystallized intelligence reflects the accumulation of knowledge, knowledge and event memory interact, and fluid intelligence and working memory correlate. Naturally, the lay public has implicit theories of these constructs. These theories mainly distinguish between intelligent and unintelligent behaviors and tend to include characteristics outside psychometric studies of intelligence, such as emotional intelligence. Here, we asked lay participants from the online platform Prolific to explain “what does being intelligent mean to you?” as well as “knowing” and “remembering” to understand their degree of alignment with theoretical conceptualizations in the research community. Qualitative coding of participant definitions showed that intelligence and knowledge are closely related, but asymmetrically—when defining what it means to be intelligent, participants reference knowledge, but intelligence is not considered in explaining *knowing*. Although participants note that intelligence is multi-faceted and related to problem-solving, there is an emphasis (in terms of frequency of mentions) on the crystallized side of intelligence (i.e., knowledge). A deeper understanding of lay participants’ mental models of these constructs (i.e., their metacognitions) is essential for bridging gaps between experts and the general public.

## 1. Lay Definitions of Intelligence, Knowledge, and Memory: Inter- and Independence of Constructs

Even if you have never seen the 1997 classic *Good Will Hunting* (Van Sant 1997), you are likely familiar with its basic premise: Will, a janitor at MIT, struggles to put his remarkable intelligence to proper use, and must overcome his traumatic past to do so. Viewers are introduced to Will’s superhuman mental abilities in a scene where he calls out a Harvard graduate student for trying to pass off ideas from an obscure history book as his own in trying to flirt with a woman at a bar. Will embarrasses the student by detailing an extensive list of authors and concepts he has likely read while forming his bland, unoriginal ideas, and concludes by pointing out the exact page of the book that the student attempts to plagiarize. This scene captures the importance we ascribe to our mental abilities, as the woman caught in the middle of this encounter is so impressed by Will’s superior talents that she approaches him to introduce herself and give him her phone number. Specifically, this scene underscores the importance of three mental abilities that are the subject of the present research: intelligence, knowledge, and memory. Furthermore, this scene illustrates some of the conceptions (and misconceptions) widely held among the general public about the functioning, connectedness, and value of these cognitive processes.

Canonical theories of intelligence, knowledge, and memory often characterize these abilities as interconnected rather than distinct constructs (Cattell 1963; Sternberg 1999; Tulving 1972), though they tend to be studied separately. Historically, the study of intelligence took off in the early 20th century with Alfred Binet’s development of intelligence testing and the application of factor analysis, most prominently associated with Charles Spearman. Much of the debate over intelligence through the years hinges on one’s definition of intelligence, and many theoretical accounts of intelligence have been proposed since then (Neisser et al. 1996). For example, Cattell (1963) distinguished between two types of intelligence: crystallized and fluid. Crystallized intelligence is defined as context-specific knowledge accumulated through one’s experiences, and fluid intelligence denotes one’s ability to solve novel problems without relying on crystallized knowledge (Cattell 1963). Bridging these constructs, Sternberg (1999) suggested that general intelligence arises through the development of expertise, such that the accumulation of crystallized, or declarative, knowledge allows one to apply this knowledge to problem-solving scenarios in the future. Sternberg’s (1985) theory further posits that there are three fundamental aspects of intelligence: analytic (i.e., academic), creative, and practical (i.e., tacit knowledge that is action-oriented and achieved independently from others’ help; Sternberg et al. 1995), though only analytic intelligence is tested in mainstream tests, and practical intelligence appears to be independent of both fluid and crystallized intelligence (Cianciolo et al. 2006). Some have argued that practical intelligence should be understood as independent of school performance or psychometric test scores (Neisser et al. 1996). For example, one can apply practical intelligence to carrying out mathematical equations required for survival in a street business and still fail mathematics in school.

Since the early days of intelligence research, Spearman and others noted that many mental ability tests were positively correlated with one another (i.e., a positive manifold; Protzko and Colom 2021), suggesting the existence of an underlying general intelligence factor, referred to as *g* (Spearman 1904). The *g* factor theory has been refined and hotly debated over the years, in part because of cultural and socioeconomic biases inherent in testing and racist overtones in how the work has been used (Alfano et al. 2016; Helms 2012). It is beyond the scope of this paper to review all theories of intelligence; rather, we focus on the relationship between knowledge, memory, and intelligence and on lay or implicit theories.

We examined how lay participants conceptualize these constructs to gain insights into their metacognitions about them. Metacognition, broadly defined, refers to “thinking about thinking” and, at an individual level, allows one to assess one’s own abilities in a given domain. Here, we posit that in order to encourage individuals to be able to effectively assess their abilities, it is helpful to know how they define or conceive of these abilities. In other words, if one has to estimate their knowledge about a given topic, it is essential to verify that they are defining knowledge or *knowing* consistently. A deeper understanding of lay conceptions is essential for dispelling myths and misinformation about these constructs. If researchers hold certain definitions of a construct that are at odds with what participants believe, the validity of individual judgments and assessments will likely be reduced (see Umanath and Coane 2020 for a discussion in the context of memory, specifically with regard to the remember/know paradigm).

Participants’ understanding of the constructs under investigation can differ from the consensus held by researchers; pre-existing conceptions of their own abilities and the ways in which cognitive processes operate can influence performance. For example, the fixed vs. growth mindset literature suggests that pre-existing beliefs about the nature of intelligence determine performance and behaviors (Dweck 1986). Metacognitive illusions—such as the Dunning–Kruger effect (Kruger and Dunning 1999) and the above-average effect (Alicke et al. 1995)—are often described as situations in which participants misjudge their abilities, generally by attributing higher levels of knowledge or skill to themselves than is warranted by their performance on a criterion task. However, it is also possible that some of these effects are influenced by what participants consider when assessing their own abilities. For example, Kim et al. (2017) found that lay definitions of “average” did not correspond to a mathematical average but reflected more common usage of the term, where “average” has some connotations of “less than good”. 

Misconceptions of memory and its accuracy can affect legal/real-world decisions. Beliefs in the accuracy of childhood memories, memories of traumatic events, or recovered memories can result in severe consequences when these memories are, in fact, fallacious (Loftus 2004, 2017; Loftus and Davis 2006). In addition, students often fail to accurately assess their own level of preparation or the validity of study strategies. Specifically, work by Kornell and Bjork (2008) suggests that students often make a metacognitive judgment that one strategy (e.g., massing vs. interleaving) is more effective than the other, whereas the opposite is often the case (see McCabe 2011 for a discussion of study strategies in undergraduate students). The persistent belief in learning styles—and how one’s preferred learning style can impact their academic performance when it is not “matched” by instructional style—illustrates how, even in the absence of supporting evidence, misinformation about how learning occurs has significant impacts on educational systems (Pashler et al. 2008). Thus, individuals’ and society’s beliefs of what is effective or ineffective, as well as the challenges involved in accurately assessing performance, can have widespread effects. These examples illustrate the importance of more clearly understanding how core constructs that are held in high regard at a societal and inter-personal level are defined outside of the confines of academia.

In fact, a point of active discussion in the field of intelligence research is how to define intelligence—and whether defining such a complex construct is even necessary or feasible (De Boeck 2013). When considering the nature of intelligence, clearly, multiple factors are involved, from genetic to environmental to cognitive and neurological. One of the challenges addressed by a number of scholars is how to interpret the positive manifold (Protzko and Colom 2021). Such correlations are likely influenced by the fact that many measures of intelligence tap into knowledge and skills that are emphasized in formal education in Western societies (Johnson 2013). In other words, scores on intelligence tests reflect exposure to the types of skills and material that are standard in education. Such interdependence leads researchers to attempt to develop “culturally neutral” measures, which, however, can never truly be abstracted from specific environmental and contextual factors—factors that are reflective of the complexity of lived environments and are rapidly changing (Johnson 2013; Protzko and Colom 2021). Given the emphasis in educational settings on creating and solidifying knowledge and the reliance on memory during learning, these three constructs are closely interconnected in real-world settings and in lay understandings. Given these challenges, to what extent do lay participants define intelligence and related constructs similarly to the experts? 

We suggest that understanding how lay participants conceptualize and understand intelligence or being intelligent and its relationship with other core cognitive constructs is essential not only for valid measurements, but also for the scientific community to accurately communicate research findings in ways that minimize distortion and misuse (e.g., the Bell Curve; Conley and Domingue 2016; Neisser et al. 1996; theory of multiple intelligences; Visser et al. 2006; White 2008). Effective communication from experts to the ultimate consumers of information depends on what the audience knows and believes (Grice 1989). Therefore, we attempt to identify the ways in which the lay public defines intelligence and core cognitive constructs related to it (i.e., knowledge and memory). In other words, we are examining what non-experts think about and how they understand these constructs, their cognitions, and metacognitions. We start by reviewing the scientific literature on how these constructs are related and then present a summary of the research examining lay understandings.

## 2. Knowledge and Memory and Their Relationship with Intelligence

Knowledge, or semantic memory, is considered part of long-term memory. Semantic memory typically consists of stored information about rote facts, symbols and their corresponding meanings, and general rules by which the world is organized—all of which are generally classified as knowledge (see Balota and Coane 2008; Kumar 2021, for reviews; Tulving 1972). In contrast, event memory, which entails a sense of remembering and a dimension of mental time travel, allows one to relive temporally dated autobiographical experiences and events (Tulving 1972; see Szpunar and McDermott 2008; Rubin and Umanath 2015). Ample evidence suggests these subtypes, although distinct, are not independent and influence one another on various tasks (see Marsh et al. 2008). For example, prior knowledge can affect performance on episodic memory tests (Coane et al. 2021; Umanath and Marsh 2014) and episodic content and context affect retrieval from semantic memory (Coane and Balota 2011). Furthermore, episodically acquired information can transfer to semantic memory over time, as evidenced by the use of ‘I remember’ when recalling information immediately after learning it and ‘I know’ when recalling the same information after a delay of several weeks (Conway et al. 1997). 

Classical theories of memory further distinguish between short- and long-term storage (e.g., Atkinson and Shiffrin 1968); more recent models propose a distinction between a working memory (WM) system, which is responsible for maintaining and processing small amounts of information for very brief delays (e.g., Baddeley and Hitch 1974; Cowan 2001), and long-term memory, which encompasses episodic and semantic memory. Importantly, memory researchers link fluid intelligence to WM, highlighting the strong correlations between g and WM and identifying shared bases for executive functions and fluid intelligence (e.g., Conway et al. 2002, 2003; Engle et al. 1999; Kane et al. 2004; but see Ackerman et al. 2005). Thus, at least within the scientific community, these three constructs are clearly related: knowledge comprises the contents of one’s semantic memory store; crystallized intelligence is understood as the accumulation of knowledge; knowledge and event memory interact to determine performance on a variety of tasks (e.g., Marsh et al. 2008); and fluid intelligence, in particular *g*, and WM tend to correlate.

These constructs often converge in their practical uses as well. For example, commonly used intelligence tests include tasks that measure crystallized and fluid intelligence separately and then form an aggregate score (Kaufman and Kaufman 1993; Wechsler 1981). Within these tasks, memory and knowledge often play a role in how each is assessed. Performance on fluid intelligence tasks, such as Raven’s Progressive Matrices (Raven et al. 1977), is directly related to WM because one must keep the mental representation of the matrix active while distinguishing between answer choices that may or may not fit (Engle et al. 1999). 

Intelligence tests often include questions for which one must draw from their base of general knowledge (i.e., semantic memory), such as the Kaufman Adult and Adolescent Intelligence Test (KAAIT; Kaufman and Kaufman 1993). Scores of intelligence tests are also affected by the fact that familiar words are recognized more easily than unfamiliar words (Hulme et al. 1991). Vocabulary, or one’s memory for words and their definitions, is a direct function of semantic memory (Tulving 1972). Because many tasks used in the KAAIT (e.g., auditory comprehension) rely heavily on verbal comprehension, vocabulary size affects performance and scores (Dunn 1987). Thus, these constructs do appear to be somewhat inter-dependent.

## 3. Lay Theories of Knowledge, Memory, and Intelligence 

The above discussions emerge from theories of memory, knowledge, and intelligence proposed by experts. However, the lay public has implicit theories of these constructs, which form individual beliefs about one’s own abilities and deficiencies. In other words, individuals have their own metacognitions not only of their own abilities, but more generally about the nature and function of these constructs. These beliefs likely guide behavior, much like other metacognitive judgments such as those discussed above (Flavell 1979; Hacker et al. 2000; Nederhand et al. 2020). Studies of such lay beliefs have been fruitful in providing insight into the nature and individual perception of these concepts. In the context of memory, misconceptions about how memory functions are widespread (Heck et al. 2018; Magnussen et al. 2006; Simons and Chabris 2011), both among the general public and among psychology students (Furnham and Hughes 2014). Much of the work examining lay theories of memory has focused on understanding how misconceptions of memory might affect legal proceedings. As noted above, if jurors and judges hold flawed beliefs about memory, such beliefs have real-life implications (e.g., Loftus and Davis 2006). Akhtar et al. (2018) identified what they termed a “common sense” memory belief system held by the lay public, which was generally inconsistent with scientific evidence (e.g., a belief that memory works like a video camera). Even individuals with training in psychology appear to hold several misconceptions (Magnussen and Melinder 2012). 

In other cases, however, lay understanding and expert knowledge align. For example, understanding the processes involved in memory retrieval from episodic versus semantic stores has been aided by phenomenological studies—specifically, the use of the terms *remember* and *know*, which according to lay participants and psychology experts alike refer to retrieval from episodic/event memory and semantic memory/the knowledge base, respectively (Umanath and Coane 2020; Coane et al. 2022; Conway et al. 1997). Similarly, lay participants and experts agree that *not remembering* and *not knowing* reflect retrieval failures due to a lack of accessibility and availability, respectively (Coane and Umanath 2019; Tulving and Pearlstone 1966; Umanath et al. 2023). Notably, however, prior work has shown that the majority of the literature does not use these terms in this way (Umanath and Coane 2020). Furthermore, it has been repeatedly demonstrated that the terms *remember* and *know* are difficult for participants to understand and use (Geraci et al. 2009; Maylor 1995; McCabe and Geraci 2009; Perfect 1996; Rubin and Umanath 2015; Strack and Forster 1995; Williams and Moulin 2015; Yonelinas 2002). Overall, these and other findings demonstrate that participants’ use of R/K can be very easily manipulated (Eldridge et al. 2002; McCabe et al. 2011; Rotello et al. 2005; Williams and Lindsay 2019; see also, Bodner and Lindsay 2003). This simply illustrates the importance of consistent alignment between participants and researchers in defining the terms and constructs of interest.

Lay theories of intelligence have also been investigated. In one of the earliest studies to assess laypeople’s views of intelligence, Sternberg et al. (1981) asked participants to list and rate behaviors associated with intelligence. They concluded that intelligent behaviors could be assigned to one of three groups: problem-solving abilities, verbal abilities, or social-competence abilities. Since Sternberg et al.’s paper, investigations of lay theories of intelligence have followed several directions. Fitzgerald and Mellor (1988) demonstrated that these theories mainly distinguished between intelligent and unintelligent behaviors, but that different intelligent behaviors did not elicit strong distinctions. As lay theories of intelligence tend to encompass human characteristics excluded from traditional psychometric studies of intelligence, such as emotional, social, and practical intelligence, other researchers have attempted to measure and elucidate these aspects of intelligence. For instance, Mayer and Salovey (1993) investigated the ways in which emotional intelligence is a unique subcategory of social intelligence. Relative to the broader construct of social intelligence, emotional intelligence is more distinguishable from general intelligence due to its distinct manifestations and underlying mechanisms (i.e., emotionality, management of emotional information, and specialized neural substrates). Cianciolo et al. (2006) examined the relationship between practical intelligence—as assessed by naturalistic measures of tacit knowledge—and general intelligence (*g*). Although everyday practical intelligence was independent from that of *g*, there was an overlap (i.e., a high–moderate correlation) between the two constructs. 

## 4. The Present Work

Although a substantial amount of work has examined lay theories of intelligence and some work has explored lay theories of memory and knowledge, there appears to be a gap in understanding how lay participants perceive these constructs to be related to one another. As noted, in psychometric testing and experimental tasks, there are clear areas of overlap, and scores on many measures used in intelligence testing are positively correlated. The questions we addressed here were how do lay participants define intelligence or being intelligent, knowledge or knowing, and memory or remembering, and to what extent do participants consider these three constructs to be inter-related? We opted not to ask participants to define the abstract constructs but the act or state of being intelligent, knowing, or remembering, because we wanted to focus on individual conceptions to tap more closely into metacognitive processes. Specifically, compared to the abstractness of the term *memory*, participants have direct experience with the act of *remembering*; thus, we posited that this wording would encourage participants to rely on experiential information. Through qualitative coding, we examined the similarities and differences across participant definitions of what it means to remember, to know, and to be intelligent. A clearer understanding of what participants mean when they consider these cognitive abilities is essential for researchers interested in how participants assess their own abilities (e.g., Chamorro-Premuzic and Furnham 2006; Furnham and Chamorro-Premuzic 2005; Furnham et al. 2001; Kruger and Dunning 1999; Rammstedt and Rammsayer 2000; Sternberg et al. 1981) to minimize concerns about measurement validity. For example, if lay participants consider knowledge to be a more central element of intelligence than experts, judgment of one’s own or other’s intelligence might reflect this, even if researchers are primarily interested in judgments of fluid intelligence. 

This work also represents a conceptual replication and extension of Umanath and Coane’s (2020) study, in which participants were asked what they meant when they said “I remember/I know”. Here, the wording changed to emphasize the action implied by the verb “what does remembering/knowing mean?” over the role of the specific agent. Examining to what extent similar dimensions emerge as a function of this variation is important for a deeper understanding of participants’ mental models of these core cognitive constructs.

### 4.1. Method

Participants. Data collection occurred at two timepoints (April 2020 and September 2020) as part of a separate task (Coane et al. under review). For ease of interpretation and because no experimental manipulations preceded the questions analyzed here, we combined the data across timepoints. Participants (n = 425) were recruited for an online survey using Prolific (www.prolific.co), an online data collection tool where participants can complete studies for monetary compensation. Participants were required to be at least high school graduates (average was 14.31 years of education, *SD* = 2.08), between 18 and 30 years old, and have a US IP address. These requirements ensured that participants’ conceptualizations of the constructs in question would be related to their common uses in American culture and that they would have a similar level of general knowledge. Sixteen participants who opened the survey revoked consent and seven timed out, leaving 402 data sets (238 women; seven unidentified). The average age was 22.90 (*SD* = 3.30, range 18–32). The average completion time for the entire study, including the portions not reported here, was 7.48 min (*SD* = 5.06, range 1.40–40.87), and participants were compensated at an average rate of $12.24 USD per hour. The study was approved by the Institutional Review Board at Colby College.

Materials and Procedure. Participants clicked on a link to the online survey. The survey was programmed and conducted via Qualtrics (Qualtrics, Provo, UT) and participants provided informed consent at the outset. The first section included three open-ended questions, in random order, where participants were instructed to type their responses: (1) “What does ‘remembering’ mean to you?”, (2) “What does ‘knowing’ mean to you?” and (3) “What does ‘being intelligent’ mean to you?” The coding of these questions is reported here. These questions were presented at the very beginning of an experimental task examining the stability of self-assessments of one’s intelligence, memory, and knowledge. Results from the experimental task are not reported here but can be found in Coane et al. (under review). At the end of the task, participants provided their age and gender and were debriefed. Payment was made directly through Prolific.

### 4.2. Results

#### 4.2.1. Response Coding

Each response was manually coded for the presence or absence of a number of dimensions: 1 = dimension present/referred to, 0 = dimension absent/not referred to. All responses were coded by two coders (SU and JHC). For all dimensions, a code of 1 indicated the dimension was mentioned, regardless of whether participants indicated a high or low level of the construct. The initial inter-rater agreement (based on the correlation between rater scores) was .98 (range .96–1.00). Discrepancies were resolved by discussion and finalized by SU. Although specific dimensions were selected separately for *being intelligent*, *remembering*, and *knowing*, each response was coded on all dimensions (coders did not know which question participants were answering except when participant responses included language such as “To me being intelligent means …)”.

The dimensions specific to *being intelligent* included theoretically important constructs and dimensions identified from the responses (see Table 1). Definitions were coded as Multi-Faceted if they referred to multiple types or forms of intelligence (e.g., emotional vs. “booksmarts”; Gardner 1987) because Multiple Intelligences Theory is often addressed in the American education system. In addition, responses were coded as Multi-Faceted if they referred to different processes or components (e.g., having knowledge and applying it). Items were coded as reflecting Application if they explicitly referred to using or applying information or one’s intelligence and as reflecting Problem-Solving if they more specifically noted that a problem or challenge needed to be addressed or solved. The dimension Acquisition referred to mentions of how a given construct is involved in the acquisition or learning of information. Given the emphasis in the lay public (and especially among educators; Bråten and Strømsø 2004; Yeager et al. 2019) on goal orientations, specifically on the difference between fixed and growth mindsets (Dweck 1986), we also examined whether participants referenced entity or incremental theories of intelligence (i.e., Mindset). The dimension of Creativity captured the use of a construct in divergent thinking, coming up with original or novel solutions, and so on. Finally, the dimension of Comparison was intended to reflect references to other individuals (e.g., being more or less intelligent than others). 

The dimensions specific to *remembering* and *knowing* were the same as those used in Umanath and Coane (2020). These consisted of a number of theoretically derived dimensions and additional dimensions that emerged from participant responses. Recollection and Familiarity refer to, respectively, the type of retrieval from memory associated with the retrieval of specific details and a sense of reliving the moment vs. a feeling of familiarity in the absence of specific details (Yonelinas 2002). The Episodic dimension reflects the mention of retrieving a specific event or episode (Rubin and Umanath 2015). As noted below, we did not code for Semantic because it was redundant with the dimension of Knowledge. Responses were coded for Accuracy if participants referred to the perceived correctness or truth of a fact, piece of information, or event and for Confidence if the response reflected some level of certainty (either high or low). Fluency referred to the ease or difficulty of retrieving a memory or performing a task and Mastery to whether there was a depth of ability or expertise. Finally, the dimension of Experience referred to the fact that information was learned or acquired through experience.

To directly examine whether participants spontaneously associate one or more of the constructs with one another, responses specific to each construct (*being intelligent*, *knowing*, *remembering*) were coded to determine to what extent participants referred to one or both of the other constructs (intelligence, knowledge, memory). For example, if participants defined *being intelligent* as including Knowledge, this was captured by these dimensions. Because we specifically coded all responses for reference to knowing/knowledge, this became redundant with the Semantic dimension used in Umanath and Coane (2020). Therefore, we only report the findings for Knowledge. See Table 1 for the complete list of dimensions and participant sample responses.

In the following analyses, we first report the overall proportion of times participants referred to each dimension and whether these proportions varied as a function of the construct being defined. Next, we examined how often participants referred to one of the other two constructs in their definitions of each individual one (e.g., references to intelligence or *being intelligent* in answering regarding *knowing* and *remembering*). Importantly, because each response was coded on all dimensions, any given response could reflect multiple dimensions; therefore, the proportions do not sum to 1. To preview our findings, we generally replicated the patterns observed in Umanath and Coane (2020) for *remembering* and *knowing*, despite framing the questions in a slightly different way here. Furthermore, *being intelligent* and *knowing* emerged as strongly, albeit asymmetrically, related: *being intelligent*, according to lay participants, includes having a large knowledge base, but *knowing* does not imply that one has intelligence.

**Figure 1:**
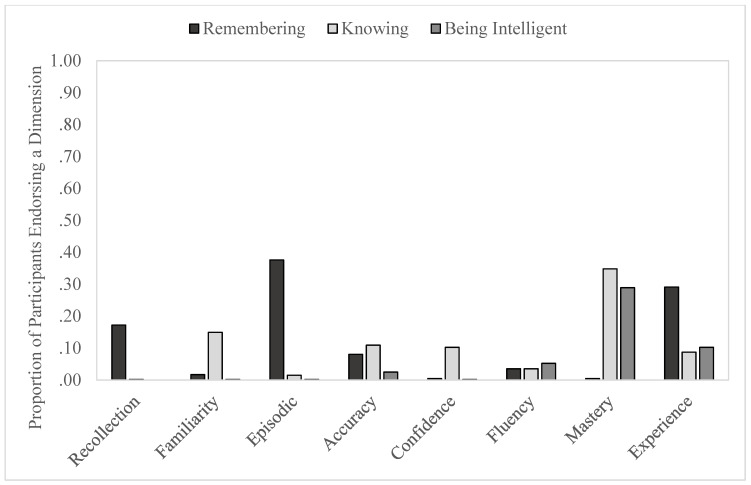
Dimensions that lay participants associate with remembering, knowing, and being intelligent.

**Figure 2:**
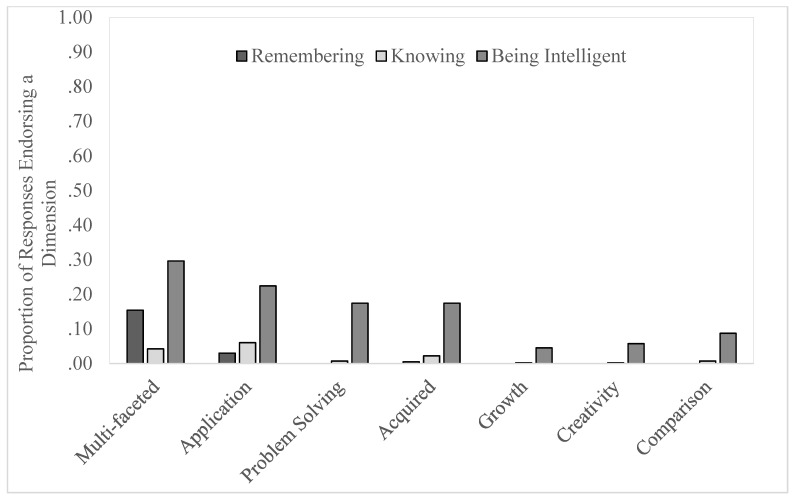
Dimensions of intelligence that lay participants associate with remembering, knowing, and being intelligent.

Several repeated measures ANOVAs were conducted to examine the relative inclusions of different dimensions in answering what *being intelligent*, *remembering*, and *knowing*, meant. For all dimensions except Fluency, the overall ANOVAs were significant. To help make sense of the findings, we organize them by the question for which each dimension was referenced most often in participants’ responses (see Figure 1 and Figure 2). Statistical statements are shown in Table 2.

##### “What Does *Being Intelligent* Mean to You?”

All the dimensions we hypothesized might be involved in defining what it means to be intelligent were indeed most referenced with regard to this question compared to the other two. Multi-Faceted was referenced most for *being intelligent* (*M* = .30), then significantly less for *remembering* (*M* = .15; most references to this dimension captured the fact that remembering involves retrieval of specific events or retrieval from the knowledge base), and finally, significantly less for *knowing* (*M* = .04). This was the only dimension for which references were significantly more frequent for defining *remembering* versus *knowing*; for all others, after *being intelligent*, there was no difference in mentions for *knowing* versus *remembering*. Application, Problem-Solving, Acquired, and Comparison all showed a pattern of reference for *being intelligent* most, *knowing* next, and mentions for what *remembering* means were lowest, although the difference between the latter two was not significant when applying a Bonferroni correction for multiple comparisons. For Growth and Creativity, again, these dimensions were most often referenced in answering what *being intelligent* means (*M_Growth_* = .05; *M_Creativity_* = .06) with no difference then between *knowing* and *remembering* because the references were essentially zero (*M*s = 0, 0, .002, .002). Notably, however, these two dimensions were not referenced all that often even for *being intelligent*. In sum, other than the dimension of Multi-Faceted, which was referenced for *remembering* as well as for *being intelligent*, the dimensions specific to *being intelligent* do appear to capture a construct that is quite distinct from the other two constructs.

##### “What Does *Remembering* Mean to You?”

Recollection, Episodic, and Experience were the dimensions that participants mentioned the most in response to being asked about the meaning of *remembering* versus *knowing* or *being intelligent*. Participants referenced Recollection for *remembering* (*M* = .17) more than for *knowing* (*M* = .003) or *being intelligent* (*M* = .00), with no difference between *knowing* and *being intelligent* (*p* = .16). More responses included Episodic for *remembering* (*M* = .38) than for *knowing* (*M* = .02) and than for *being intelligent* (*M* = .003); the latter two did not differ. Finally, for Experience, the same pattern emerged as for Recollection. That is, participants referenced Experience for *remembering* (*M* = .29) more than for *knowing* (*M* = .09) or *being intelligent* (*M* = .10), with no difference between the latter (*p* = .22). Note that the patterns regarding *remembering* and *knowing* with regard to Recollection and Episodic are consistent with the findings of Umanath and Coane (2020) in which participants were asked to define what it means to say “I remember” and “I know”. Interestingly, in that previous work, Experience was more associated with “I know” than “I remember”. We address this discrepancy in the Discussion.

##### “What Does *Knowing* Mean to You?”

The dimensions that participants mentioned the most in response to being asked about the meaning of *knowing* compared to *remembering* and *being intelligent* were Familiarity, Confidence, and Mastery. Participants referenced Familiarity for *knowing* (*M* = .15) more than for *remembering* (*M* = .02) and for *being intelligent* (*M* = .003). Confidence was referenced almost exclusively for *knowing* (*M* = .10) and not *remembering* (*M* = .01) or *being intelligent* (*M* = .002), with no difference between *remembering* and *being intelligent* (*p* = .28). Regarding Mastery, though it was mentioned most for *knowing* (*M* = .35), it was also included with some frequency for *being intelligent* (*M* = .29), whereas it was almost never referenced for *remembering* (*M* = .01). Because we did not code the previously used Semantic dimension, we examined the references to Knowledge in response to *knowing*: the majority of responses (.73) included a reference to knowledge, confirming that the act of *knowing* does appear to reflect retrieval from the knowledge base. Note that the patterns regarding *remembering* and *knowing* are consistent with those in Umanath and Coane (2020), with all three of these dimensions being more associated with *knowing* than *remembering*.

#### 4.2.2. Other Patterns

##### Accuracy and Fluency

References to Accuracy showed a unique pattern. That is, this dimension was mentioned similarly often for both *remembering* (*M* = .08) and *knowing* (*M* = .11) and significantly less so in reference to *being intelligent* (*M* = .03). This pattern lies in contrast to that found in Umanath and Coane (2020) in that Accuracy was referenced significantly more often for defining “I know” than “I remember”. Perhaps this construct is one for which “knowing” and “I know” are different in participants’ conceptualizations of knowledge. 

As mentioned above, the overall ANOVA was not significant for Fluency, indicating that there were no statistically significant differences among references to this dimension across the three questions. Similar to Umanath and Coane (2020), this dimension was simply not referenced very frequently in general with the means being .04, .04, and .05 for what *remembering*, *knowing*, and *being intelligent* mean, respectively. 

#### 4.2.3. References to Memory, Intelligence, and Knowledge in Responses to the Questions

Memory was mentioned more often when participants were defining *knowing* (*M* = .16) than *being intelligent* (*M* = .01), *t*(401) = 2.99, *p* = .003, *d* = .15. Knowledge was referenced significantly more frequently in explaining what it means to *be intelligent* (*M* = .49) than *remembering* (*M* = .41), *t*(401) = 2.42, *p* = .016, *d* = .12. Additionally, Intelligence was rarely included when participants defined *remembering* (*M* = .005) or *knowing* (*M* = .01), *t*(401) = .82, *p* = .415. Taken together, Memory has something to do with how we consider the construct of *knowing*, and Knowledge is definitely involved in how we explain *remembering* and *being intelligent*, but surprisingly, Intelligence has little to do with how we conceptualize *remembering* or *knowing*.

## 5. Discussion

The qualitative coding of participant responses revealed areas of agreement and disagreement between lay participants’ conceptualizations and the scientific community’s conceptualizations of these three core constructs. Below, we first discuss the findings for each construct and areas of overlap and separation, then the perceived relationships between them, and conclude by discussing how these findings can fit into the broader field of intelligence research. 

### 5.1. Views on Being Intelligent

Existing theories of intelligence posit that crystallized intelligence is comprised of general knowledge (Cattell 1963), and general knowledge is often used as a measure of crystallized intelligence and is included on standardized tests. Thus, experts seem to agree that knowledge is a valid measure of intelligence to some extent, though a holistic measurement of intelligence must include measures of fluid intelligence (e.g., Kaufman and Kaufman 1993; Wechsler 1981). Previous lay theories of intelligence (e.g., Sternberg et al. 1981), in contrast, highlight the relative importance of practical intelligence, or what Sternberg and colleagues referred to as “everyday intelligence”, with less emphasis on crystallized intelligence or knowledge. Prior evidence suggests that more naturalistic measures of tacit knowledge to assess practical intelligence (i.e., college undergraduate living, entry-level workplace positions, and everyday practical situations) in a sample of American adults revealed relatively small differences between general intelligence and practical intelligence (Cianciolo et al. 2006). The two types of intelligence (practical vs. general) were still recognized as distinct, but there was more of an overlap between them when measures were more naturalistic and less specialized.

Here, the dominant dimension in defining what it means to *be intelligent*, referenced by almost half of the participants, was Knowledge. This underscores the extent to which, in the layperson’s view, the distinction between crystallized and fluid intelligence may be less relevant or important than it is to scholars in the field. One could argue that utilizing one’s knowledge is similar to “everyday intelligence”—in other words, the use of information or experiences to serve specific goals and enable behaviors to achieve those goals. In fact, a common theme in participant responses was that *being intelligent* was about applying or using the knowledge one has—in other words, intelligent behaviors (as possibly captured by the use of the phrase *being intelligent*) involved the active integration of information already possessed by the individual and the situation or challenge at hand. In their work, Sternberg et al. (1981) found that Knowledge, as reflected by the item “is knowledgeable about a particular field of knowledge”, was loaded onto a general verbal ability factor in lay participants (see their Table 4, pg. 45). However, for experts, knowledge as stored information did not appear to be a clear dimension (see their Table 5, pg. 46). Experts considered a “good vocabulary” an important dimension in the Verbal Intelligence factor whereas the ability to “apply knowledge to problems at hand” was a dimension in the Problem-Solving Ability factor. Our results align rather closely with this apparent difference between experts and non-experts: lay participants attribute more importance, at least explicitly, to knowledge than experts appeared to do in earlier work. We acknowledge experts’ definitions were not collected in the present work, so our conclusions are tentative in this regard. 

The other two most commonly referenced dimensions in the context of *being intelligent* were Mastery and Multi-Faceted. Participants associated *being intelligent* with levels of deep knowledge, expertise, and a broad skill set. Specifically, a number of participant responses referenced having a “strong understanding” or “knowing a lot about different subjects”. Additional common responses included mentions of “understanding” or “comprehension”. Thus, Mastery in the context of *being intelligent* appears to be in part tied to Knowledge and in part about the ability to understand information and situations easily and comprehensively. 

The dimension of Multi-Faceted captured a variety of participant responses. Many participants did reference different forms or types of intelligence, such as those proposed by Gardner’s (1987; Gardner and Hatch 1989; see also Mayer and Salovey 1993) Multiple Intelligences (e.g., spatial, kinetic, artistic). This may represent a disconnect between what lay people believe about intelligence and what scholars consistently support. Though many find this theory appealing, the assessment tools to adequately measure each type of intelligence proposed remain unclear (Visser et al. 2006; White 2008), and there are semantic arguments to be made about whether Gardner’s proposed intelligences are just that—intelligence or talents (Reisberg 2021; White 2008). Furthermore, Gardner proposed that these different intelligences are distinct and fairly independent rather than representing aspects of intelligence as a whole (Sternberg and Sternberg 2016), which may or may not be how lay people understand the theory. Note, however, that Multi-Faceted was also used to identify responses in which participants mentioned several, overlapping functions of intelligence, such as problem-solving and having high knowledge, which also occurred frequently. Thus, this dimension was coded more broadly than examining only multiple intelligences and also captured the fluid vs. crystallized distinction made by scholars. In this context, the frequent reference to Multi-Faceted may indicate that *being intelligent* seems to be the “glue” that binds together multiple cognitive processes (attention, speed, problem-solving, etc.).

It is also worth noting that overall, participants made very few references to Creativity, which emerged as a strong dimension among experts in the Verbal Ability factor in Sternberg et al. (1981). In addition, hardly any participants explicitly referred to aspects of *being intelligent* that mapped onto the distinction between entity (fixed) and incremental (growth) mindsets (Dweck 1986). Given the widespread nature of this concept and the extent to which mindsets have been examined as predictors of academic success (Costa and Faria 2018; Mangels et al. 2006), this finding was somewhat unexpected. It is possible that the framing of the question did not lead participants to consider such aspects—considering what *being intelligent* means might prime participants to think more about specific behaviors or attitudes, rather than broader frameworks, conceptualizations, or characteristics of intelligence itself. Whether such results would emerge were the question framed differently remains an open question. Alternatively, mindset differences might be less salient to participants unless they are explicitly asked about them.

In sum, lay participants have rich and complex understandings of what constitutes *being intelligent*. These metacognitions likely influence behaviors—if one considers Knowledge as a core component of *being intelligent*, then seeking out information or being perceived as possessing high levels of Knowledge likely influences how one’s intelligence is perceived (Coane et al. under review). However, Knowledge alone is clearly not sufficient for defining intelligent behaviors. As evident in the distribution of responses, being intelligent might be akin to “Knowledge Plus”: having, using, and applying one’s knowledge to solve problems and acquire more information seems to capture what lay participants express.

We acknowledge that the present work was conducted from a Western perspective with Western-residing participants. A rich body of literature has examined lay theories of intelligence across cultures. For example, Cocodia (2014) found that Eastern/Asian, Western, and African lay conceptions of intelligence all generally emphasize cognitive abilities, knowledge, and social skills. However, culture and intelligence are intertwined, so different cultures’ conceptions of intelligence have unique characteristics. For example, Asian cultures tend to emphasize religious and philosophical elements, whereas rural African communities tend to prioritize practical and social abilities integral to performing routine tasks. Additionally, even within a single culture, there are subcultures that have distinct values, and thus, unique notions of intelligence (Cocodia 2014). 

Thus, whether our findings would extend to more diverse samples and to groups with different cultural backgrounds remains an open question. There do appear to be differences in implicit theories of intelligence across cultures (Cocodia 2014). For example, Ishida et al. (1991) examined implicit theories of intelligence among people from Japanese, Korean, Chinese, Taiwanese, Canadian, and Mexican cultures. Although the importance of factors among Asian cultures was similar and included five dimensions (sympathy and sociability, interpersonal competence, ability to comprehend and process knowledge, accurate and quick decision making, and ability to express oneself), they differed compared to Canadian and Mexican cultures. Taiwanese concepts of intelligence overlapped with similar concepts identified in studies conducted in the United States but showed emphases on practical/contextual aspects of intelligence, which is most similar to older rather than younger US adults (Yang and Sternberg 1997). In contrast, Lim et al. (2002) conducted a study on Korean implicit theories of intelligence and suggested that while cultural differences exist, they tend to be differences of degree rather than of kind. Most notably, when participants were asked to rate hypothetical profiles for intelligence, less emphasis was placed on social competence—a characteristic that distinguishes Korean implicit theories from American ones. Thus, clear cultural differences exist in consideration of these cognitive constructs. The present work can only address and is limited by the participants’ cultural environment, and it is unclear whether similar relationships between the three constructs examined here would manifest outside the US. Future work should certainly explore this.

### 5.2. Views on Remembering and Knowing

A secondary aspect of the present work was to replicate and extend previous research examining the way in which lay participants define the classic memory paradigm terms “remember” and “know”. Our findings add to understanding the differences between *remembering* and *knowing* (Tulving 1985; Umanath and Coane 2020). Previously, lay participants were asked “what do you mean when you say I know/remember?” In common use, *remembering* and *knowing* reflect markedly different phenomenological states, such that the statement ‘I remember’ is associated with the retrieval of event-related information from event memory, and the statement ‘I know’ is associated with retrieval from semantic memory. Here, participants were asked to respond to “what does remembering/knowing mean to you?” Although this difference may seem subtle, it does reflect a difference in orientation for thinking about these concepts and their underlying constructs. 

In several ways, the present results showed the generalizability of the terms. The three dimensions that most strongly captured *remembering* were Recollection, Episodic, and Experience. Replicating prior work, *remembering*, therefore, does seem to capture, in the layperson’s mind, the act of mental time travel, where one retrieves a specific prior event or learning episode and can recall or recollect specific details. The dimensions that were referenced most frequently for *knowing* were Familiarity, Confidence, and Mastery, although the former two were quite low overall (less than .15). This suggests that, although there are some aspects of *knowing* that capture that feeling of Familiarity and Confidence, the strongest association is that of Mastery: when lay participants define *knowing*, they describe it as reflecting a deep understanding or “really knowing it”.

However, there were also differences from Umanath and Coane (2020). For *remembering*, interestingly, Knowledge was mentioned more than any other dimension, suggesting that *remembering* also involves the retrieval of information from the knowledge base. In other words, participants seem to consider *remembering* as an action—the act of retrieval, regardless of whether the to-be-retrieved information is event-based or knowledge-based. As noted above, this could reflect the subtle difference between “I remember” and “remembering”. Why might this small change matter? Specifically, *I remember* may refer more to individual acts of recollection, whereas *remembering* may encompass a general ability to remember, retain, and retrieve information. Likewise, *I know* may be associated more with individual acts of retrieval from the semantic memory base, whereas *knowing* may refer to a broader or more static trait that applies to multiple domains and situations. 

References to the dimensions of Experience and Accuracy also appear to differ from earlier work. For Experience, Umanath and Coane (2020) found that it was mentioned more for *I know* than for *I remember*; here, the opposite was observed. A closer examination of the results from Umanath and Coane, however, indicated that the difference in usage frequency was driven by the expert participants; lay participants did not significantly differ in their usage of the term, although it was in the same direction as experts. It is also possible that the wording change from *I remember* to *remembering* created subtle interpretation changes in participants. The other dimension that appeared to be at odds with earlier work was Accuracy—in Umanath and Coane, it was a very frequently used dimension for *I know*; here, it was used less often when defining *knowing*, indeed no differently than for *remembering*. One possibility is that the emphasis shifted more to Mastery, which emerged as a central dimension (in fact, here, Mastery was present in almost 40% of responses compared to 20% in the previous work). Colloquially, individuals might use *I know* in the context of “I know this to be true/factual” and *knowing* might be referring more to a somewhat static state of “that which is known or stored in memory”. We acknowledge this explanation is speculative at this point. Regardless, these inconsistencies underscore the importance of asking participants what they mean when commonly used terms are used to tap into scientific constructs, and they highlight the potential variations that can occur with even relatively minor wording changes (see also, Williams and Lindsay 2019; McCabe et al. 2011).

For *knowing*, a number of responses also referenced Memory. In line with how *remembering* reflected retrieval from the knowledge base, participants somewhat frequently (.16) referred to the fact that knowledge was stored in memory or memory banks. These findings fit in with existing theories positing that remembered information becomes knowledge through repeated exposures and schematization (Schank and Abelson 1995) and with research demonstrating that people first *remember* information and can trace it to the event where it was learned, but later claim to *know* the information after memory for the initial encoding event is lost (Conway et al. 1997). 

The broader field of epistemological beliefs examines the nature of knowledge and the process of knowing. Some of the core dimensions that multiple models of epistemological beliefs share (see Hofer and Pintrich 1997 for a review) are that knowledge can be absolute or relative, that it is handed down by experts or constructed by the learner, and that knowledge can include uncertainty. Knowledge further can evolve from the accumulation of facts to the development of a richly connected network of related concepts. Knowing can be defined in terms of two dimensions: the source (e.g., self vs. experts) and the justification (e.g., authority vs. evidence). Our dimension of Mastery appears to align with the justification dimension, in that once a level of expertise is achieved, there is a reduced need to justify one’s knowledge. Interestingly, in the context of how we queried participants, we found very few references to the acquisition process or the role of experts or authority in the development of knowledge. In fact, the dimension most relevant for the acquisition would be the Experience dimension, which was relatively low when participants were defining *knowing.*

One point made explicitly by Hofer and Pintrich (1997) in their review was that views on intelligence, although they might have implicit influences on learning, are best kept separate from the study of epistemology. Our findings suggest that this might be possible when studying beliefs about knowledge, in that participants hardly ever referenced intelligence, but that, when defining *being intelligent*, participants frequently mentioned Knowledge and *knowing*. Therefore, lay theories of intelligence do appear to include some elements of epistemological beliefs.

### 5.3. Implications and Recommendations

In the field of memory research, there has been an active discussion about certain tasks used by researchers to measure core cognitive processes that might be prone to error or misunderstanding on the part of participants. For example, the Remember/Know paradigm (Gardiner and Java 1991; Rajaram 1993; Tulving 1985) has been used for decades to decompose recognition memory into two distinct processes: recollection and familiarity. A growing body of research, however, suggests that the specific wording of the instructions given to participants (e.g., *remember* vs. *recollect*; *familiar* vs. *know*) alters the estimates of the underlying processes (e.g., McCabe and Geraci 2009; Williams and Lindsay 2019). A number of researchers have also noted that using terms such as *remember* and *know*, which have well-established meanings outside of a laboratory context, can create unique challenges for certain populations, such as individuals with amnesia (e.g., Aggleton et al. 2005) or older adults (McCabe and Geraci 2009). Migo et al. (2012) proposed a series of recommendations for improving the administration of the remember/know paradigm, as did Umanath and Coane (2020); these included using standardized instructions, assessing compliance, and verifying comprehension on the part of participants. A simple recommendation we provide here is that researchers consider asking their participants to define key terms employed in a study, regardless of whether the researchers themselves provided definitions of these terms. Verifying that the terms were used consistently across participants and within participants over testing sessions can reduce instances of disconnect between the scholarly community and lay participants. 

Beyond the use of specific terms used in research contexts, both lay individuals (Magnussen et al. 2006) and psychology experts (Magnussen and Melinder 2012) endorse a number of statements and beliefs about memory that are not empirically supported. Even among the latter group, consisting of individuals with doctoral degrees in the field of psychology, knowledge about memory revealed a number of errors and misconceptions. Many of these misconceptions can have profound effects in legal and court settings. 

In the field of intelligence, misconceptions among lay individuals relative to expert understandings of the construct are quite common. For example, Warne and Burton (2020) reported that a large number of teachers and non-teachers endorsed beliefs about intelligence that were at odds with expert consensus. Especially among the former group, they noted such misconceptions have the potential to impact how educators interact with learners and the extent to which they might recommend interventions or recommend children for gifted programs. Furnham and Horne (2021) reported similar findings, showing that a large number of participants endorsed “intelligence myths”—statements about intelligence that are not strongly supported by empirical evidence or expert consensus.

Thus, the work presented here adds to an existing body of work showing the importance of understanding how these constructs—especially memory and intelligence—are understood by lay participants. Unfortunately, correcting such misinformation appears to be quite challenging: even experts are not immune to some misconceptions (Magnussen and Melinder 2012; but see Simons and Chabris 2011), and the growing literature on correcting misinformation suggests this is a challenging task (see Lewandowsky et al. 2012, for an extensive discussion). 

### 5.4. Limitations and Future Directions

We note that our approach is heavily qualitative in nature; our interest is in determining participants’ own understandings of these constructs, rather than identifying underlying factors or systematic relationships between dimensions and constructs or addressing the behavioral consequences of holding some particular dimensions versus others as part of one’s definition of these related constructs. Clearly, such approaches are valid and important; they are, however, outside of the scope of the present paper. Exploring more quantitative or experimental approaches is a clear avenue for future research. Additionally, as noted, we did not consult experts—in part because one natural consequence of expertise in one domain or construct does not imply expertise in the other constructs. For example, not every intelligence researcher is also going to be an expert in memory and vice versa, thereby rendering a definition of “expert” challenging. Finally, as noted above, this work is grounded in Western approaches to understanding the constructs under investigation; whether such definitions and relationships would emerge in other cultural settings remains open to investigation. 

## 6. Conclusions

To conclude, the present research demonstrates that it is critical to examine the ways in which lay people conceptualize cognitive constructs such as *being intelligent*, *knowing*, and *remembering*. Currently, a substantial focus of scholarly research in intelligence is on fluid intelligence, the psychometric testing of intelligence, and the biological and neurological bases of intelligence. A recent Special Issue of the journal *Intelligence*, devoted to the future of research in the field, focused on five core areas: measurement issues, neurological and genetic components and contributions, education, and artificial intelligence (Haier 2021). Almost a decade earlier, a Special Issue of the *Journal of Intelligence* identified similar key areas when considering the most important issues in the field; experts agreed on the importance of studying the neural processes and the importance of measurement precision in interventions to raise intelligence at the population level (Hunt and Jaeggi 2013). Although there is some discussion on how to define intelligence and research on what myths and misconceptions of intelligence and memory are held among lay participants, in an era in which information and misinformation are readily available, one important step is understanding what beliefs are held by the lay public. Naïve psychological science (Taylor and Kowalski 2004) includes many misconceptions about these and other constructs. In this context, metacognition is important because individuals need to be aware of discrepancies between their beliefs and actual facts and adopt strategies to overcome those discrepancies.

In closing, a 1921 symposium asked leading scientists of the field to define intelligence and describe how it could be best measured. The opinions were quite diverse, although there was a general consensus that (1) intelligence is difficult to define, and (2) it represents something that is at least partially innate and therefore distinguishable from knowledge, which is acquired. Such findings appear to remain valid 100 years later (De Boeck 2013; Haier 2021) and apply to lay participants as well as to experts.

## Figures and Tables

**Table 1:** Coding dimensions, definitions, and participant sample responses.

Dimension	Definition	Example Participant Response
DIMENSIONS SPECIFIC TO “REMEMBERING” AND “KNOWING”		
Recollection	Response includes reference to Recollection of specific details or uses word recollect	Being able to reflect on a time in your past and feel the specific emotions or senses associated with that moment
Familiarity	Response notes “feels familiar” or response indicates a lack of detail combined with a sense of prior experience/mention of “awareness”	Having a memory that is accompanied by feelings of familiarity, but lacks specific details
Episodic	Response indicates retrieval of specific event from the past	Recalling facts, images, scenarios and being able to picture these things in your mind
Accuracy	Response includes reference to perceived accuracy of retrieved information (includes statements such as “true”, “factual”, “evidence-based”)	Being able to accurately recall information.
Confidence	Response includes reference to confidence or certainty of answer	To be certain of a fact, thought, or idea.
Fluency	Response includes statements that reflect the ease of retrieval, the speed/automaticity with which information comes to mind	To have information in your head intuitively. It is there, you do not need to do anything to recall and use it
Mastery	Response indicates depth of understanding or mastery of material	Knowing means that you have internalized and understand the material. When talking about a subject that you know it means you can expand upon the subject and go into detail about it.
Experience	Response includes a reference to the fact that the information was acquired through learning or prior experience	Knowing is the result of successful learning.
DIMENSIONS SPECIFIC TO “BEING INTELLIGENT”		
Multi-Faceted	Response refers to multiple types/forms/facets/aspects of the construct, from many sources	Having a knowledge of events, books, life events. Having wisdom. Being emotionally intelligent.
Application	Response refers to using or applying information or knowledge	Knowing many things without reference and using them in ways that are beneficial to you
Problem-Solving	Response indicates importance of construct for solving problems	Being capable of using the knowledge you have in a critical and interpretive manner
Acquisition	Response indicates its importance for learning/acquiring new information	Being intelligent means being able to pick up concepts and ideas quickly and having the ability to apply them.
Mindset	Response refers to fixed or growth mindset/innate/genetic	Having the genetic ability to learn fast.
Creativity	Response refers to thinking outside the box, using information in new/unusual ways	Applying one’s knowledge in untraditional ways
Comparison	Response includes some form of comparative judgment relative to others	Knowing more information than those around you.
OTHER CONSTRUCTS MENTIONED		
Memory/ remembering	Response given refers to memory or remembering	To have an extraordinary problem-solving ability that draws from a large store of knowledge via a quick and accurate memory
Knowledge/ knowing	Response given refers to knowledge or knowing	Being able to learn and retain knowledge with minimal effort.
Intelligence	Response refers to intelligence or being smart	Knowing is a bit like a combination of both intelligence and memory; it’s both being able remember something and having the ability to use/explain that information.

**Table 2:** Summary of statistical analyses.

	Effect of Construct	*p*-Value	Effect Size	Pairwise Comparisons
Dimension				
Recollection	*F*(1.03, 411.58) = 81.22	<.001	.17	R > K; R > I; K = I
Familiarity	*F*(1.22, 490.09) = 54.28	<.001	.12	K > R; R > I; K > I
Episodic/Event	*F*(1.10, 445.30) = 219.77	<.001	.35	R > K; R > I; K = I
Accuracy	*F*(1.90, 762.41) = 12.40	<.001	.03	R = K; R > I; K > I
Confidence	*F*(1.12, 450.04) = 81.22	<.001	.09	K > R; R = I; K > I
Fluency	*F*(1.94, 778.22) = 1.09	.337	.00	3
Mastery	*F*(1.72, 690.27) = 98.33	<.001	.20	K > R; I > R; K = I
Experience	*F*(1.84, 736.40) = 49.07	<.001	.11	R > K; R > I; K = I
Multi-Faceted	*F*(1.81, 725.56) = 56.87	<.001	.12	R > K; I > R; I > K
Application	*F*(1.50, 600.70) = 53.96	<.001	.12	R = K; I > R; I > K
Problem-Solving	*F*(1.08, 432.33) = 79.03	<.001	.16	R = K; I > R; I > K
Acquisition	*F*(1.27, 509.38) = 62.90	<.001	.14	R = K; I > R; I > K
Growth/Mindset	*F*(1.08, 436.62) = 16.79	<.001	.04	R = K; I > R; I > K
Creativity	*F*(1.07, 427.84) = 22.24	<.001	.05	R = K; I > R; I > K
Comparison	*F*(1.13, 453.19) = 32.00	<.001	.07	R = K; I > R; I > K

Notes: The reported measure of effect size is partial eta squared. Pairwise comparisons are significant at *p* ≤ .017 (to account for multiple comparisons).

## Data Availability

The studies were not pre-registered. Data are available at: https://web.colby.edu/memoryandlanguagelab/publications/stimuli-and-data-sets/.
